# Characteristics of Idiopathic Sensory Processing Disorder in Young Children

**DOI:** 10.3389/fnint.2021.647928

**Published:** 2021-04-28

**Authors:** Shelley Mulligan, Sarah Douglas, Caitlin Armstrong

**Affiliations:** ^1^Department of Occupational Therapy, University of New Hampshire, Durham, NH, United States; ^2^Department of Communication Sciences and Disorders, University of New Hampshire, Durham, NH, United States

**Keywords:** sensory processing disorders, diagnostics, adaptive behavior, clinical presentation, evaluation

## Abstract

This study described the developmental and behavioral characteristics of children identified with idiopathic sensory processing disorder (SPD) as well as the relations among specific types of SPD as proposed by the nosology presented by Miller et al. (2007), adaptive behavior profiles, and behaviors associated with mental functioning. A retrospective, non-experimental design applying descriptive and correlational analyses was used. Data were obtained from clinic medical records of 78 children ages 2 to 7 years who were identified with sensory processing problems affecting daily life, but who did not meet criteria for any other neurodevelopmental or mental disorders following a comprehensive diagnostic evaluation. Results revealed that all SPD types as described by current typologies were well represented with the most common being the over-responsivity sensory modulation subtype. Within the sample, 53% of the children displayed more than one SPD type. Atypical externalizing and internalizing behavior scores associated with various mental disorders as measured by the child behavior checklist (CBCL) fell in the borderline dysfunctional range. Adaptive behavior for all developmental domains was below average, and the severity of SPD symptoms moderately and positively correlated with behaviors associated with mental disorders, and with lower adaptive behavior performance. It was concluded that symptoms characteristic of the various types of idiopathic SPD overlap substantially suggesting that current typologies may include more types/subtypes than are necessary or clinically useful. Children with SPD share similar, but often less severe pathological behaviors associated with other mental or related neurodevelopmental disorders. Psychometrically sound measures of SPD are needed, and further study of the neural mechanisms involved in sensory processing deficits is vital for validating idiopathic SPD as its own diagnostic entity.

## Introduction

Sensory processing and integration are complex neurodevelopmental functions allowing children to regulate, perceive, discriminate, and use sensory input experienced from the environment and internally from their bodies to effectively respond, learn, and adapt throughout daily life. Sensory processing disorder (SPD) has been described as a distinct neurodevelopmental disorder in the literature (Schoen et al., 2009; Jorquera-Cabrera et al., 2017; Crasta et al., 2020) and has been recognized as its own diagnostic entity in the most recent version of the diagnostic classification of mental and developmental disorders of infancy and early childhood-revised (DC: 0–5, zero to three). Sensory processing abilities develop naturally and play important roles in child learning, behavior and emotional regulation, motor development, and task performance. Sensory processing disorder has simply been defined as the brain’s inability to organize sensory input for appropriate use. As stated in the DC: 0–5, zero to three manual, SPD is diagnosed based on the presence of difficulties in detecting, modulating, interpreting or organizing sensory stimuli to the extent that these deficits impair daily functioning and participation. However, the question of whether deficits in sensory processing represent symptomology of another disorder such as autism spectrum disorder (ASD), or developmental coordination disorder (DCD), or SPD is its own distinct condition remains (Borkowska and Sklodowska, 2017). The aim of this study was to examine and describe the developmental and behavioral characteristics and profiles of children identified with idiopathic sensory processing disorder (iSPD). Relations among specific types and subtypes of SPD, adaptive behavior and psychosocial-emotional functioning were also examined to further our understanding of how childhood neurodevelopmental and mental disorders sharing similar symptomology may be distinguished from SPD, and how children with iSPD fit within the current SPD typology (Miller et al., 2007).

The American Academy of Pediatrics [AAP], 2012 recommended that SPD generally should not be diagnosed citing that there is no universally accepted framework for the SPD diagnosis. Although sensory processing problems were recognized as being important to identify and address in children with a variety of neurodevelopmental conditions, the AAP stated that there lacked evidence to solidly demonstrate that children presenting with sensory-based problems have an actual “disorder” of the sensory pathways of the brain. It was concluded that sensory processing deficits were likely associated with other developmental and behavioral disorders. This view, however, does not account for children who present with sensory processing deficits affecting their daily life who do not meet diagnostic criteria for any other disorder. Such children may be referred to as having iSPD.

A typology developed in a seminal paper by experts in the field characterized SPD as having three main types: (a) sensory modulation disorder (SMD); (b) sensory discrimination disorder (SDD); and (c) sensory-based motor disorder (SBMD), which are further divided into subtypes (Miller et al., 2007). SMD includes three subtypes; sensory over-responsivity (SOR), sensory under-responsivity (SUR), and sensory craving (SC; sometimes referred to sensory seeking) and considers the visual, auditory, tactile, vestibular, proprioception, gustatory, olfactory sensory systems, as well as interoception. Sensory modulation refers to the ability to notice and react to, regulate, adapt to, and grade responses that are appropriate to the sensory situations experienced in daily life. Atypical behaviors associated with SOR are characterized by intense, exaggerated responses to sensory events that most children do not perceive as negative or noxious often resulting in withdrawal, avoidance behavior. Atypical behaviors with SUR include muted or slowed responses to sensory experiences often with an apparent lack of awareness, lethargy and/or indifference, or diminished responsivity. Finally, atypical behavior associated with SC includes a need for more intense sensory input than what one would normally want or that would naturally occur often manifesting as inappropriate, disruptive, disorganized and/or risky behavior (Miller et al., 2007, 2017; James et al., 2011; Schoen et al., 2014).

Sensory discrimination disorder refers to problems with the ability to accurately perceive and interpret sensory information coming in or experienced from one or more of the sensory systems (Miller et al., 2007; Lane and Reynolds, 2020; Parham and Mailloux, 2020). Discrimination abilities allow for the recognition of qualitative and quantitative sensory features and differences among various objects and experiences processed through the sensory systems. People with this type are capable of registering sensory stimuli, however, appreciating or detecting the qualities of a given stimulus is a challenge and can occur with one or more sensory systems. Finally, SBMD has two subtypes, postural disorder, and dyspraxia. Postural disorder consists of problems with stabilization of the body during movement or at rest, diminished motor strength, balance, and coordination. Dyspraxia involves impairments in planning motor sequences and performing new motor activities with difficulties with coordination, and synchronizing movements, and learning and performing new motor tasks (Miller et al., 2007; Lane and Reynolds, 2020; Parham and Mailloux, 2020).

Based on a review of epidemiological studies, prevalence estimates of iSPD ranges from 5 to 16% of children in the general population, while 60–90% of children with coexisting neurodevelopmental conditions such as autism spectrum disorder (ASD) have been estimated to have sensory problems (Ahn et al., 2004; Ben-Sasson et al., 2009; James et al., 2011; Galiana-Simal et al., 2020; Jussila et al., 2020). Mulligan et al. (2019) examined the psychometrics of a new measure of sensory processing and reported that 20% of the children in their sample had a co-occurring disorder while the remaining 80% had not been diagnosed with another neurodevelopmental disorder. SPD was identified based upon the global clinical impression of experienced occupational therapists following a comprehensive evaluation including standardized motor tests, parent report behavior scales and interviews, and a performance-based sensory processing measure. Unfortunately, there are a limited number of diagnostic assessment tools available to evaluate the sensory processing abilities of children, and no tool available for specifically identifying and measuring the SPD types as described in the typology by Miller et al. (2007). The Sensory Integration and Praxis Test (Ayres, 1989) is a norm-referenced, performance-based assessment that has long been the gold standard for measuring sensory integration and praxis in children 4 to 9 years of age, although it does not measure sensory modulation. Other available tools for measuring sensory processing functions are caregiver report behavior rating scales including the Sensory Processing Measure (Parham et al., 2007) and the Sensory Profile-2 (Dunn, 2014). These tools measure sensory processing according to sensory systems, and the Sensory Profile-2 also provides scores for specific sensory processing patterns including sensory seeking, low registration, sensory sensitivity, and sensory avoiding. A new performance-based test, the Sensory Processing Three Dimensions Assessment largely based upon the SPD diagnostic typology has recently been developed, and will soon be available for clinical use (Mulligan et al., 2019).

Research evidence is building regarding how sensory processing deficits manifest within various populations, as well as how iSPD may be differentiated among children with other neurodevelopmental conditions. As high as 90% of children with ASD have sensory processing difficulties with the most common pattern of SPD being the SOR modulation subtype (Schoen et al., 2009; Tavassoli et al., 2014; Tomchek et al., 2014; Little et al., 2018). Children with ASD were found to be more under-reactive to auditory stimuli, but over-reactive to taste and smell. Behaviors or symptoms associated with a variety of mental health conditions such as depression, and anxiety have also been reported in children with SPD with higher rates of atypical internalizing and externalizing behaviors including inattention, hyperactivity, mood disturbances, and anxiety (Engel-Yeger et al., 2016; McMahon et al., 2019; Van Hulle et al., 2019). Sensory under-responsivity has been associated with depressive symptoms and internalizing behaviors, while externalizing behaviors have been more associated with sensory craving, and over-reactivity. Studies demonstrating how the symptoms of children with iSPD differ from those seen in children with other neurodevelopmental conditions including ADHD and ASD are particularly relevant for supporting SPD as its own diagnostic entity. Miller et al. (2012) compared clinical assessment findings of among samples of neurotypicals, children with ADHD, those with SMD, and those with dual diagnoses. All clinical groups had significantly more sensory, attention, activity, impulsivity, and emotional difficulties than typical children. However, inattention was greater in children with ADHD compared to SMD, and children with SMD had more sensory issues, somatic complaints, anxiety/depression, and difficulty adapting than those with ADHD. Moreover, children with SMD had greater physiological/electrodermal reactivity to sensory stimuli than the ADHD group, and neurotypicals. Schoen et al. (2009) compared the profiles of children with SMD, with typically developing children, and those with ASD. They found that physiological arousal and sensory reactivity were lower in children with ASD. Although both clinical groups had significantly more sensory-related challenging behaviors than the typical children, the clinical profiles differed. The ASD group had more taste/smell sensitivity and sensory under-responsivity, while the SMD group had more atypical sensory seeking behavior. Tavassolia et al. (2018) examined whether children with ASD show more sensory symptoms and different cognitive styles in empathy and systemizing as compared to children with SPD, and typically developing children. Of interest was that a finding across groups, was that greater severity of sensory symptoms was associated with lower empathy. As expected, both autism and SPD groups showed more sensory symptoms than neurotypical children and the ASD group showed more sensory under-reactivity than the SPD group. Together, the results of these studies suggest that ADHD, ASD, and SMD- a type of SPD, are distinct conditions.

Evidence is also growing for supporting SPD as its own distinct disorder due to unique underlying neuropathology or neural substrates within sensory pathways of the brain. Symptoms often seen in SMD for example, have been explained by a lack of gating function in the basal ganglia (Davies and Gavin, 2007; Davies et al., 2009; Chang et al., 2014). The basal ganglia has a role in selective attention, and when not working optimally may result in poor gating such that a child may not notice salient environmental sensory information, or experience difficulty filtering out irrelevant sensory input. EEG recordings compared children with SPD to typically developing children and discovered that children with SPD had less auditory sensory gating capabilities. Moreover, they found that among typically developing children, sensory gating abilities increased with age, while these abilities did not advance among children with SPD. Brain processing differences have also been found among neurotypical children, children with ASD, and children with SPD (Chang et al., 2014; Demopoulos et al., 2017). ASD and the SPD groups showed white matter pathology in the sensory processing regions of the dorsal visual stream, and significant differences in white matter were noted between the neurotypicals and those with SPD (Owen et al., 2013). Researchers also found that neural pathways for social emotional functioning-the fusiform gyrus connections to the hippocampus and amygdala were significantly affected in the children with ASD, but not in the children with SPD, or neurotypicals (Chang et al., 2014). These pathways are known to process emotions based upon facial expression, a problem commonly described as a feature of ASD. Not surprising were findings that the most extensive white matter alterations in the SPD group were seen in the parieto-occipital tracts which are responsible for auditory, tactile, and visual perception and integration of sensory input. Abnormalities within the cerebro-cerebellar system may contribute to sensory hypo- and hyper- responses because of the cerebellum’s role in detecting the level of intensity of experienced sensory stimuli. Damage in certain areas of the cerebellum have been found to contribute to atypical sensory processing as well as motor problems (Koziol et al., 2011). Children with SPD symptoms were also found in this study to have imbalance of Purkinje cells in the cerebellum, with increased number of Purkinje cells resulting in sensory hypo-responsiveness, while too few leading to hyper-responsiveness. A study examining children born with agenesis of the corpus callosum (AgCC) found reduced sensory registration among participants with AgCC as compared to typical children especially with auditory processing (Demopoulos et al., 2015). The authors concluded that further understanding of the sensory processing patterns among people with AgCC may lead to clarification of the role that the corpus callosum plays in SPD. Larger gray matter volumes have been shown to be associated with atypical sensory processing related to taste and smell, touch, and the processing of visual and auditory stimuli (Yoshimura et al., 2017). Furthermore, neuroimaging of children identified specifically with auditory over-responsivity were found to have white matter tracts showing decreased fractional anisotropy relative to children without sensory over-responsivity (Tavassoli et al., 2019). Collectively this research is beginning to show that SPD has distinct neuropathology within the sensory pathways of the brain.

Research examining the occupational performance and adaptive behavior of children have well-documented that sensory processing abilities impact a child’s performance across developmental and functional domains. Social participation and play skills have been found to be negatively affected as well as performance in activities of daily living including self-care, eating/mealtime routines and problems with sleep (Koenig and Rudney, 2010; Dunn et al., 2016; Williams et al., 2018). School-related problems are also documented as a consequence of sensory processing deficits include lower academic achievement, inattention in the classroom, learning difficulties and motor challenges which impede performance of activities like handwriting, and participation in physical education (Ashburner et al., 2008; Chien et al., 2016). The relatively high prevalence, and detrimental effects of SPD on the daily lives of children necessitate efforts to advance our knowledge in the areas of diagnostics and intervention for this condition.

This study explored the developmental and behavioral characteristics and profiles of children who did not meet criteria for any neurodevelopmental condition, but who were identified as having symptomology relating to one or more of the types of SPD as described by Miller et al. (2007). Relations among specific types and subtypes of SPD, adaptive behavior and psychosocial-emotional functioning were examined as a step toward understanding the developmental profiles of these children, and to assist in understanding how other neurodevelopmental and mental disorders sharing similar symptomology may be distinguished from SPD.

## Materials and Methods

A retrospective, non-experimental design applying descriptive and correlational analyses was used. Assessment data were obtained from an existing data set that had been extracted and compiled from the records of children seen at a clinic for a developmental and/or diagnostic evaluation from 2014 to 2017 in the Northeastern United States. The children ranged in age from 1 to 7 years of age, and had been evaluated by an interdisciplinary team of professionals including a developmental pediatrician, occupational therapist, speech-language pathologist, and others as needed such as an early education specialist, physical therapist, and social worker. Data were extracted from the clinic medical records by a trained research assistant following procedures that were approved by the Institution’s Internal Review Board for Protection of Human Subjects of the first author.

Inclusion criteria for the cases selected for analyses were that the child: (a) be between 2 and 7 years of age; (b) have SPD symptoms based on scores from the Sensory Profile (Dunn, 2014) with at least 2 sensory processing area scores greater than 1 standard deviation (SD) from the mean or one area greater than 2 SDs from the mean; (c) no documented or reported neurodevelopmental or mental disorder such as ASD, or intellectual disorder affecting neurodevelopment. Children with significant visual and auditory impairments were excluded. Children with unspecified motor, communication, or cognitive delays were included as long as delays in development were not associated with a known neurodevelopmental, intellectual, or mental disorder. Assessment data from 58 males and 20 females (*N* = 78) ranging in age from 24 to 70 months met criteria and were included. Mean age was 46.5 months (SD = 11), with most children (65%) ranging in age from 3 to 5 years. The children were primarily from Caucasian families (78.5%) while the remainder reported race as being African American (3%), Mixed (6%), Hispanic (4%), Asian (5%). All were referred for a comprehensive developmental assessment because of developmental delays, behavioral concerns, and/or a specific question relating to a possible ASD diagnosis. Those children who were confirmed to have a diagnosis including ASD following the comprehensive assessment were excluded.

### Measures

#### Sensory Profile, Sensory Profile-2 (Dunn, 2014)

Factor and Area scores from the child version (sensory profile-2 or sensory profile), or infant/toddler version were available and used to identify the children with iSPD. Sensory Profile scores were examined and transformed so that the types of SPD noted in the SPD typology, and processing problems for specific sensory systems could be rated as absent-0, some indication but mild presentation-1, and definite presentation-2. A rating system was necessary and developed for the purposes of this study since the Sensory Profile is based upon Dunn’s model of sensory processing (Dunn, 2014) which differs somewhat from the way in which SPD types and subtypes are categorized within the typology by Miller et al. (2007). Dunn’s model and the Sensory Profile versions provide norm-referenced scores for 4 distinct sensory processing patterns: Sensory Avoiding and Sensory Sensitivity which both reflect behaviors associated with sensory over-responsivity, sensory registration which is associated with sensory under-responsivity, and sensory seeking which reflects sensory craving behaviors. Five types of SPD were identified including Miller et al.’s three sensory modulation subtypes-SOR, SUR, and SC; SBMD, and SDD. Based on the Sensory Profile items and data available, it was not possible to distinguish between the two subtypes of SBMD defined by Miller et al., Postural Disorder and Dyspraxia so scores related to either subtype were considered under SBMD. To illustrate, the Sensory Profile-2 (Dunn, 2014) provides scores for Sensory Avoiding and Sensory Sensitivity patterns that are reflective of the SOR pattern. Therefore, if either one of those patterns were scored between 1 and 2 SD from of the mean (interpreted as a probable difference), then the child was rated as a 1 (a mild or probable concern) for the SOR SPD type. If a child scored more than 2 SD from the mean for the Sensory Seeking Profile (interpreted as a definite difference), than they were rated a 2 for the sensory craving SPD type. The specific criteria used to assign ratings for the 5 SPD types are provided in the Supplementary Appendix. Sensory areas for visual, auditory, tactile, and vestibular/proprioception (movement and body awareness) were rated using the same 0-1-2 scale, with a 0 (typical) assigned when a Sensory Profile sensory area score fell within the typical range, 1 (mild problem) when the area score fell between 1 and 2 standard deviations from mean, and 2 (definite problem) when scores fell 2 or more standard deviations from the mean. Sensory Profile data for taste and smell, and interoception are limited and therefore these sensory areas were not included in the analyses (refer to criteria used to assign ratings for specific sensory system processing in the Supplementary Appendix). Finally, to obtain an overall SPD severity score, the ratings (0, 1, or 2) from each of the five SPD types and four sensory system areas were summed so that higher scores (max = 18) indicated a more severe clinical presentation of iSPD. Reliability of the diagnostic ratings was established by using two independent raters who assigned the ratings for the SPD types based on the Sensory Profile item ratings, factor, and area scores. Inter-rater agreement of SPD type ratings was 86% with disagreements occurring only for the SDD and SBMD types. Where disagreements occurred between ratings, the two researchers discussed the ratings and reached consensus based on applying the *a priori* criteria as presented in the Supplementary Appendix.

#### Child Behavior Checklist (CBCL; Achenbach and Ruffle, 2000)

The CBCL is part of the Achenbach system of empirically based assessment and is a caregiver report measure of externalizing and internalizing behaviors organized by 8 syndrome scales such as anxious/depressed, withdrawn/depressed, and attention problems. The CBCL has been shown to have acceptable reliability, validity and predictability with actual mental disorder diagnoses. Standard scores from child behavior checklist (CBCL; T-scores, mean = 50, SD = 10) for externalizing behaviors, internalizing behaviors, and total behaviors were examined along with T-scores for each of the scales associated with symptomology from a disorder as defined by the Diagnostic and Statistical Manual of Mental Disorders IV-TR: affective, anxiety, oppositional defiant, pervasive developmental, and attention hyperactivity. T-scores falling 1.5 standard deviations above the mean (65 or greater) were interpreted as in the mild/probable dysfunctional range; scores 70 and above represent a definite indication of behaviors associated with the disorder.

#### Vineland Adaptive Behavior Scales-2nd Edition (VABS-2; Sparrow et al., 2005)

The VABS-2 is a norm-referenced caregiver questionnaire for measuring adaptive behavior within the domains of communication, social, daily living, and motor skills. Standard scores from VABS-2 for each of the four domains and their respective subdomains were used for analyses, along with the adaptive behavior composite score. Caregivers rate their child on items using a 3-point scale representing the frequency with which the child performs a specific skill or behavior. Studies support the VABS-2 as a valid and reliable measure of adaptive behavior (Sparrow et al., 2005). Domain and composite standard scores of 1 standard deviation or more below the mean are interpreted as being significantly below average or in the dysfunctional range.

Data were analyzed using SPSS version 24, and included descriptive statistics, and Spearman-*r* correlations to examine relations between types and subtypes of sensory processing disorders, and developmental, and behavioral variables. The non-parametric Spearman correlation was selected as the sensory processing, developmental, and behavioral variables being correlated were derived from ordinal scales.

## Results

### Sensory Processing Disorder Types

All five SPD types explored were well represented in the sample as follows: sensory over-responsivity (SOR; 60%), followed by under-responsivity (SUR; 51%) and sensory craving (42%). Sensory-based motor disorder was identified in 40% of the sample, and 29% were identified with sensory discrimination disorder (SDD). A notable finding was that 53% of the sample experienced more than one SPD type. Of those, the most common combination was children with all five types (16/41, 39%), which might be interpreted as global or generalized SPD, while 30% exhibited all three types of sensory modulation disorder (SOR, SUR, and craving) without showing sensory discrimination problems or SBMD. Results examining sensory systems revealed that the tactile and proprioceptive/vestibular systems were most often affected with 76.4% of children showing some dysfunction, followed by auditory (70.6%) then visual (54.9%). The mean sensory processing severity score was 10.2 (SD = 5.6) with most children having involvement in more than one sensory system, and experiencing more than one SPD type.

### Behavior Profiles

Mean standard T scores from the CBCL were examined by SPD type, as well as the frequency (% cases) scoring in the dysfunctional range (SD = 1.5 above the mean). Mean T-scores for externalizing behaviors, internalizing behaviors, and total behaviors were borderline (65, 64, 67, respectively) with slightly more problems with externalizing behaviors (see Table 1). For all three sensory modulation types, children on average scored in the dysfunctional range for externalizing behaviors, and for the pervasive developmental disorder (PDD) scale. The mean affective disorder scale score fell in the dysfunctional range for children identified with SUR. For children identified with SDD and SBMD, mean scores were in the dysfunctional range only for externalizing behaviors and the PDD category. The behavior profiles across the SPD types were strikingly similar, with slightly more problem behaviors noted with children with SBMD. It is important to note that many children were identified with more than one type of SPD which may account for some of the similarity observed in the behavior profiles. Having a large proportion of the subjects exhibiting multiple SPD types reduced the ability to uniquely characterize the behavioral characteristics of each SPD type.

**TABLE 1:** Child Behavior Checklist T-scores by sensory processing disorder type.

CBCL Atypical Behavior Scales	Sensory craving *N* = 32			Over-reactive *N* = 46			Under-reactive *N* = 38			Sensory discrim *N* = 22			Sensory-based motor *N* = 30			TOTAL *N* = 75		
						
	*M*	SD	% Dys	*M*	SD	% Dys	*M*	SD	% Dys	*M*	SD	% Dys	*M*	SD	% Dys	*M*	SD	% Dys
Externalizing	**73.3**	10.1	36	69.1	13.6	27	**70.8**	12.2	27	**74.3**	11.3	29	**73.3**	11.2	40	65.2	13.8	25
Internalizing	67.9	10.9	20	68.1	10.1	14	68.6	10.1	15	**71.3**	10.0	18	69.0	10.5	28	63.8	11.2	16
Total	**73.5**	10.4	36	**71.4**	12.4	22	**73.3**	11.1	24	**75.9**	11.9	23	**74.7**	10.6	36	67.0	12.9	23
Affective	67.7	10.5	13	66.3	11.2	14	68.8	10.5	13	68.8	10.0	13	69.0	10.2	24	63.7	10.9	2
Anxiety	66.3	12.7	13	67.0	12.5	11	67.4	12.7	13	69.5	12.4	6	68.0	13.4	12	62.5	12.2	1
PDD	**73.9**	8.6	42	**74.2**	9.2	36	**75.1**	8.6	38	**76.5**	8.4	31	**75.7**	**7.1**	44	**70.9**	9.5	40
ADHD	67.4	7.9	46	64.1	9.1	31	65.5	8.5	28	69.2	8.0	38	67.3	8.2	44	62.4	9.2	29
ODD	68.7	8.2	33	66.2	10.2	28	66.8	9.8	28	68.8	8.5	25	68.4	8.6	40	63.8	10.3	25

*CBCL, child behavior checklist; Discrim, discrimination; PDD, pervasive developmental disorder; ADHD, attention deficit hyperactivity disorder; ODD, oppositional defiant disorder. Values bolded are 2 SD or more above the mean showing a dysfunctional pattern of behavior.*

Mean CBCL behavior scores of children were also similar regardless of the specific sensory systems affected (see Table 2) with children with tactile processing deficits showing slightly more atypical behaviors (Total behavior mean = 75.8, SD = 10.3) than those with visual, auditory, and/or vestibular/proprioceptive processing problems. The PDD behavioral profile was evident in all children regardless of the specific sensory system(s) involved.

**TABLE 2:** Child Behavior Checklist T-scores by sensory system deficit.

Atypical Behavior Scales	Visual *N* = 17			Auditory *N* = 31			Tactile *N* = 31			Vestibular/Proprioception *N* = 38			Total *N* = 75		
					
	*M*	SD	% Dys	*M*	SD	% Dys	*M*	SD	% Dys	*M*	SD	% Dys	*M*	SD	% Dys
Externalizing	**71.9**	14.6	71	**71.9**	12.5	68	**73.8**	12.0	77	69.9	11.1	66	65.2	13.8	48
Internalizing	68.7	12.1	59	69.3	9.5	61	**70.4**	9.1	68	65.8	11.6	53	63.8	11.2	47
Total	**73.9**	13.9	82	**73.3**	11.3	77	**75.8**	10.3	84	**70.8**	11.2	71	67.0	12.9	55
Affective	68.2	11.9	65	67.4	11.1	61	**70.2**	9.5	68	65.5	10.4	53	63.7	10.9	45
Anxiety	67.0	12.8	47	66.5	12.0	52	67.7	12.5	56	65.0	13.5	45	62.6	12.2	37
PDD	**74.9**	8.3	94	**74.3**	9.1	90	**76.7**	7.4	93	**73.3**	8.5	89	**70.9**	9.5	79
ADHD	67.1	8.8	65	65.9	8.9	58	67.7	8.2	61	65.7	7.9	55	62.4	9.2	41
ODD	67.1	10.3	65	67.7	9.4	58	63.9	9.7	52	66.3	8.7	58	63.8	10.3	45

*Values bolded are 2 SD or more above the mean showing a dysfunctional pattern of behavior.*

Moderate and significant positive correlations (all *p* < 0.001) were found between the SPD severity rating score and each of the mental disorder scales of the CBCL ranging from *r* = 0.44 to 0.59 (Total score) and showing that as SPD severity increased, more problem behaviors occurred. Specific correlations between the SPD severity rating with each disorder scale were: Oppositional defiant disorder *r* = 0.44, affective disorder *r* = 0.45, pervasive developmental disorder *r* = 0.50, attention deficit disorder *r* = 0.52, internalizing behaviors *r* = 0.49, and externalizing behaviors *r* = 0.57.

### Adaptive Behavior

In comparison with normative data, adaptive behavior mean standard scores fell in the dysfunctional, moderately low range for children within each SPD type for all four developmental domains: Communication, daily living, social, and motor (Table 3). Based on the VABS-II composite mean, children with SDD had the poorest performance (mean = 77.5, SD = 10.8) while children with SOR showed the highest mean score (80.7, SD = 8.9). As a percentage, more children with SBMD had an adaptive behavior composite score in the dysfunctional range than any other SPD type. However, patterns of adaptive behavior scores across the different SPD types were very similar.

**TABLE 3:** Vineland Adaptive Behavior Scales-II mean domain standard scores (*M* = 100, SD = 15) and subdomain scaled scores (*M* = 15, SD = 5).

Adaptive Behavior Scale Domains	Sensory craving *N* = 32			Over-reactive *N* = 46			Under-reactive *N* = 40			Sensory discrimination *N* = 22			Sensory-based motor *N* = 30			Total *N* = 77		
							
	*M*	SD	% Dys	*M*	SD	% Dys	*M*	SD	% Dys	*M*	SD	% Dys	*M*	SD	% Dys	*M*	SD	% Dys
Communication	82.2	11.5	47	82.4	11.7	50	81.4	11.6	60	79.2	12.2	59	81.0	11.0	53	82.0	13.3	55
Receptive	10.4	2.1	69	10.6	2.0	65	10.4	2.0	73	10.3	2.1	68	10.2	2.0	73	11.0	2.3	60
Expressive	12.7	2.7	25	12.7	2.8	28	12.5	2.8	33	11.9	3.3	32	12.8	3.0	30	12.1	3.3	39
Daily living	81.1	12.1	56	83.2	12.0	50	82.6	10.9	53	79.6	11.6	64	81.1	11.9	57	84.7	11.4	49
Personal	11.0	2.5	63	11.0	2.5	63	10.9	2.3	65	10.3	2.2	73	10.8	2.5	70	11.2	2.4	57
Domestic	12.5	2.4	47	13.2	2.7	33	12.8	2.4	35	12.5	2.5	41	12.8	2.7	40	13.6	2.6	23
Community	12.6	12.1	34	12.8	2.4	33	12.9	2.3	30	12.6	2.2	32	12.4	2.2	37	12.9	2.3	27
Social domain	82.3	3.2	56	83.4	12.0	50	81.4	11.4	60	81.0	13.7	60	81.7	12.5	60	83.7	12.5	51
Interpersonal	12.3	3.2	34	12.0	2.7	39	11.6	2.8	50	12.1	3.6	36	11.7	3.3	47	12.4	2.8	40
Play/Leisure	10.4	2.1	69	12.9	2.5	59	10.5	2.3	68	10.3	2.1	68	10.6	2.2	63	11.3	2.6	56
Coping skills	13.1	2.4	28	13.2	2.7	30	13.1	2.5	28	12.7	2.7	41	13.1	2.6	33	13.7	2.7	23
Motor domain	83.1	12.3	63	84.6	12.2	54	82.8	12.5	63	81.7	14.2	64	82.1	13.4	63	85.1	12.2	55
Gross	12.1	2.6	47	12.3	2.5	44	12.0	2.4	45	11.7	2.7	55	12.0	2.8	50	12.8	2.4	34
Fine	12.4	2.5	31	12.7	2.6	30	12.3	2.8	40	12.2	2.7	41	12.1	2.7	43	12.4	2.6	40
Composite	79.3	9.9	66	80.7	9.4	61	79.2	9.2	68	77.5	10.8	68	78.7	9.8	70	81.6	10.4	62

*Mean domain area scores are expressed as standard scores with scores falling between 85 and 115 (*M* = 100, SD = 15) interpreted as being within the average range; subdomain scores are expressed as scaled scores (*M* = 15, SD = 5) with scores falling between 10 and 20 interpreted as being within the average range.*

Scores for expressive language were slightly higher than receptive language scores, and this held true for all SPD types. For the motor domain, fine and gross motor scores were very similar across all SPD types with the exception of children with SDD, who showed slightly more gross motor problems. Children with SBMD had relative strengths with communication, and scored lowest for motor skills, while children with SUR had a relative strength in the motor area, with the lowest performance in the area of play/leisure. Adaptive behavior areas scored lowest regardless of SPD type included receptive language, play/leisure, and personal self-care skills. Relative areas of strength included coping skills within the social domain, and domestic activities within the daily living domain. Similarly, as noted above the large proportion of children having more than one SPD type reduced the ability to uniquely characterize the specific adaptive behavior strengths and weaknesses associated with each SPD type. Adaptive behavior performance deficiencies were noted regardless of the sensory system impacted. The results indicated that children with visual processing deficits had the lowest overall adaptive behavior scores. Upon examination of the adaptive behavior domains, communication was the area most impacted by children with auditory, visual and/or tactile processing problems, and motor scores were most impacted for those with vestibular/proprioceptive sensory processing deficits.

Correlational analyses showed that as SPD severity increased, adaptive behavior scores tended to decrease as would be expected. Significant, but relatively mild, negative correlations (*p* < 0.05) were obtained between the SPD severity score and receptive communication (*r* = −0.30), domestic daily living (*r* = −0.37), social play/leisure (*r* = −0.24), social community (*r* = −0.24), and gross motor (−0.31). Correlations between sensory processing abilities and adaptive behavior, however, were not as strong as the correlations between sensory processing and atypical behaviors associated with childhood mental disorders.

## Discussion

These results begin to provide a clinical presentation of young children with iSPD. There was a tremendous amount of overlap of symptoms seen in children across the various types of SPD examined, leading one to question whether the current typology of SPD (Miller et al., 2007) might be flawed with more defined types and subtypes than actually exist or are clinically useful. In support of this idea, a more refined model of sensory integration and processing dysfunction applying relevant research over the past 40 years was presented by Bundy and Lane (2020). This model identified two main types: sensory-based dyspraxia (SBD) and sensory modulation disorder (SMD). Inadequate sensory processing within the central nervous system (all sensory systems considered) was depicted by Bundy and Lane as a problem with sensory “reactivity” which results in SMD, and/or a problem with sensory perception and discrimination leading to SBD. The model further divides SMD into two subtypes: sensory over-responsivity and sensory under-responsivity similarly to Miller et al.’s typology. Sensory craving, the third SMD subtype included by Miller et al., was not included in Bundy and Lane’s model but rather excessive, sensory seeking behaviors characteristic of this pattern were viewed as symptoms of either sensory over or under-reactivity. Bundy and Lane’s model includes two subtypes of SBD, vestibular bilateral integration and sequencing, and somatodyspraxia similar to the two SBMD subtypes described by Miller et al. (2007).

Earlier research by Mulligan (1998) also presented a more refined categorization of sensory processing deficits with four patterns: dyspraxia, visual perceptual deficit, somatosensory deficit, and bilateral integration and sequencing disorder (BIS). This model was based on confirmatory factor analyses using large sample of scores of children on the Sensory Integration and Praxis Tests (SIPT; Mulligan, 1998) and was later supported by a similar study also using SIPT scores (Mailloux et al., 2011). In contrast to the other two models discussed above, Mulligan (1998) did not include sensory modulation as a type as the SIPT does not have the capacity to measure sensory modulation behaviors. Similar to Bundy and Lane, and the typology by Miller et al., Mulligan (1998) identified two sensory-based motor disorder types naming them Dyspraxia, and BIS (similar to postural disorder). Mulligan’s (1998) somatosensory deficit pattern and visual perceptual pattern captured sensory discrimination deficits but only for these two sensory systems.

To summarize, previous research aimed at examining patterns of sensory integration and processing deficits or SPD types and subtypes, along with the results from this study depict SPD as a multi-dimensional neurodevelopmental condition with a number of patterns or types. Furthermore, the patterns or types that have emerged across studies have been similar, and have highly correlated with one another with overlapping symptoms, suggesting the presence of one overreaching, or unifying construct which could be construed as iSPD. The sensory profiles of children in this study revealed that the most common pattern of SPD was the sensory over-responsivity modulation subtype despite many children showing all three subtypes of sensory modulation disorder. Perhaps it is plausible to disregard the existence of SMD subtypes and rather view over-reactivity, under-reactivity, and sensory craving behaviors as common symptoms with over-responsivity being the most defining, or prevalent characteristic. Regardless of SPD type, or the sensory system(s) involved, internalizing and externalizing atypical behaviors, along with behaviors associated with mental disorders such as anxiety, and inattention were frequently present and positively correlated with severity of sensory processing deficits. Atypical externalizing behaviors such as aggression, tantrums, and hyperactivity commonly occurred the children with iSPD and research has shown that sensory experiences processed by children with SPD often result in undesirable behaviors that project outwardly (Little et al., 2018). Sensory processing difficulties in childhood have also been shown to be predictive of an anxiety disorder later in life which may account for the presence of some of the anxious behaviors documented in the sample (McMahon et al., 2019). Although children with iSPD often share the same kinds of behaviors associated with anxiety, mood and attention disorders, child symptoms in this study typically did not reach the thresholds necessary for meeting diagnostic criteria for those other conditions.

The growing body of research examining the connections between ADHD, developmental coordination disorder (DCD), and SPD in particular presents a diagnostic dilemma as these disorders share many hallmark characteristics such as inattention, motor disorganization, and hyperactivity (Little et al., 2018). When evaluation data provides clear evidence of underlying sensory processing problems especially related to modulation and dyspraxia, and the child also has symptoms that approach the threshold for an ADHD or a DCD diagnosis, SPD may better explain the child’s clinical presentation. The way in which a child’s behaviors are interpreted for diagnostic purposes is vital because such labels provide guidance for clinical decision making regarding service delivery, types of intervention approaches, and for applying evidence-based practices.

Adaptive behavior across functional domains was impacted by the sensory processing deficits of the children and as the severity of SPD increased, adaptive behavior decreased. Social participation was a relative strength, highlighting a difference from what is typically seen in children with ASD. Findings were consistent with the abundance of literature documenting that children with SPD are less likely to participate, perform within age expectations, and derive enjoyment from their daily life activities and occupations even when study samples have included children without coexisting conditions (Chien et al., 2016; Williams et al., 2018). Moreover, specific types of SPD are being linked to certain types of occupational performance deficits such as sensory hyper-responsivity being predictive of lower performance in activities of daily living (Crasta et al., 2020).

Study limitations include the relatively small sample restricted to children ages 2 to 7 years from a single clinic in the Northeastern United States, and from primarily White, middle class families limiting generalizability to the SPD population as a whole. The use of scores from versions of the Sensory Profile to identify the children with SPD types was also a significant limitation. This tool was used because there is no reputable standardized diagnostic measure to classify the different types/subtypes of SPD as described by Miller et al.’s (2007) typology, and it was the tool used as part of the comprehensive developmental evaluation where the study took place. It is therefore plausible that the SPD type assignment may have been inaccurate for some children. Furthermore, the SOR pattern may have occurred more frequently in the sample as a function of tool which emphasizes the detection of SOR type aversive and avoidance sensory behaviors more so than behaviors associated with sensory discrimination, or sensory-based motor differences. The severity score was limited as well as it was largely based upon the number of sensory systems involved, and how many SPD types a child presented with. It is plausible that some children could have very severe symptoms associated with only one sensory system or one SPD type, and the severity of their condition would not have been captured accurately by the measurement system devised for this study. Finally, creating distinct behavior profiles based on CBCL and VABS scores for each SPD type was limited because a large proportion of the sample exhibited multiple SPD types.

An important next step for future research is the development of psychometrically sound, performance-based instruments, and standard procedures for diagnosing SPD and its various types and subtypes. The Sensory Processing Three Dimensions Assessment (SP3D) is in the final phases of development and will soon be available for clinical use (Mulligan et al., 2019). A recent study concluded that the SP3D was a helpful tool for assisting with the phenotyping sensory modulation disorders (Tavassoli et al., 2019). Research aimed at studying brain mechanisms involved in the sensory processing of children with iSPD and comparing brain processing of sensory input with that of children with related neurodevelopmental and mental health conditions is needed (Tavassoli et al., 2019). An understanding the differences in neurophysiology and neural mechanisms between children with and without iSPD, and among children who meet criteria for other neurodevelopmental and mental disorders is vital for supporting SPD as its own diagnostic entity.

## Data Availability Statement

The raw data supporting the conclusions of this article will be made available by the authors, without undue reservation.

## Ethics Statement

The studies involving human participants were reviewed and approved by the Institutional Review Board for the Protection of Human Subjects, University of New Hampshire. Written informed consent for participation was not required for this study in accordance with the National Legislation and the Institutional Requirements.

## Author Contributions

SM provided oversight and implemented all aspects of the study including assisting with the review of literature, research methods, data collection and analysis, and the writing of the manuscript. SD and CA assisted in data collection, review of literature, data collection and analysis, and writing of the manuscript. All authors contributed to the article and approved the submitted version.

## Conflict of Interest

The authors declare that the research was conducted in the absence of any commercial or financial relationships that could be construed as a potential conflict of interest.

## Supplementary Material

Supplementary A Assignment of sensory processing disorder type ratings based on sensory profile (SP) scores.
