# Supplementary material for: Characteristics of Idiopathic Sensory Processing Disorder in Young Children
Source: Front Integr Neurosci. 2021 Apr 28;15:647928. doi: 10.3389/fnint.2021.647928 (PMC8113623; doi:10.3389/fnint.2021.647928)
Supplement: Supplementary A — Assignment of sensory processing disorder type ratings based on sensory profile (SP) scores. [file Data_Sheet_1.PDF]

Appendix

Assignment of Sensory Processing Disorder Type Ratings based on Sensory Profile (SP) Scores

SPD Rating	Sensory Over-responsivity	Sensory Under-responsivity	Sensory Craving	Sensory Discrimination Disorder	Sensory-based Motor Disorder
0-pattern not present or typical	SP score typical (within 1 SD from mean) for Sensory Avoiding and Sensory Sensitivity patterns	SP in the typical range (within 1 SD) for the Sensory Registration pattern	SP in the typical range (within 1 SD from mean) for Sensory Seeking	SP sensory system area scores[1] all fall within the typical range (within 1 SD from mean)	SP scores in the typical range (within 1 SD from mean) for area scores relating to movement, and body position [2]
1-mild, or some indication pattern is present	SP score in the probable difference range (1-2 SD greater than mean) for either Sensory Avoiding or Sensory Sensitivity patterns	SP in the probable difference range (1-2 SD greater than mean) for Sensory Registration	SP in the probable difference range (1-2 SD greater than mean) for Sensory Seeking	A SP sensory system area score within the probable or definite difference range AND rating of one item suggesting a possible sensory discrimination problem	SP score in the probable difference range for one area relating movement and body position
2-definite indication pattern is present	SP score in the definite difference range (more than 2SD from mean) for Sensory Avoiding and/or Sensory Sensitivity OR SP score in the probable difference range for both Sensory Avoiding and Sensory Sensitivity patterns	SP in the definite difference range (more than 2 SD greater than mean) for Sensory Registration pattern	SP in the definite difference range (more than 2SD greater than mean) for Sensory Seeking	A SP sensory area score falling within the probable or definite difference range AND the rating of more than one item within the area suggesting a possible sensory discrimination problem	2- more than one score in the probable difference range in areas relating to movement and body position, or at least one score falling the definite difference range

[1] SP Sensory System Area Scores: Touch (tactile), Visual, Auditory, Movement or Vestibular, Body Position, Multisensory

[2] SP scores related to SBMD: Vestibular Processing, Movement, Body Position, Sensory Processing Related to Endurance/Tone, Modulation Related to Body Position and Movement
